# Systematic review of the clinical outcomes of pneumonia with a penicillin-group resistant pneumococcus in respiratory and blood culture specimens in children in low- and middle-income countries

**DOI:** 10.7189/jogh.12.10004

**Published:** 2022-08-22

**Authors:** Maeve Hume-Nixon, Ruth Lim, Fiona Russell, Hamish Graham, Claire von Mollendorf, Kim Mulholland, Amanda Gwee, ARI Review group, Trevor Duke, Hamish Graham, Steve Graham, Amy Gray, Amanda Gwee, Claire von Mollendorf, Kim Mulholland, Fiona Russell, Maeve Hume-Nixon, Saniya Kazi, Priya Kevat, Eleanor Neal, Cattram Nguyen, Alicia Quach, Rita Reyburn, Kathleen Ryan, Patrick Walker, Chris Wilkes, Poh Chua, Yasir Bin Nisar, Jonathon Simon, Wilson Were

**Affiliations:** 1 Department of Paediatrics, University of Melbourne, Melbourne, Australia.; 2 Infection and Immunity Theme, Murdoch Children’s Research Institute, Royal Children’s Hospital, Parkville, Victoria, Australia.; 3 Royal Children’s Hospital Melbourne, Flemington Road, Parkville, Victoria, Australia; 4 Department of Infectious Disease Epidemiology, London School of Hygiene and Tropical Medicine, London, United Kingdom

## Abstract

**Background:**

*Streptococcus pneumoniae* is one of the most common bacteria causing pneumonia and the World Health Organization (WHO) recommends first-line treatment of pneumonia with penicillins. Due to increases in the frequency of penicillin resistance, this systematic review aimed to determine the clinical outcomes of children with pneumonia in low- and middle-income countries (LMICs), with penicillin-group resistant pneumococci in respiratory and/or blood cultures specimens.

**Methods:**

English-language articles from January 2000 to November 2020 were identified by searching four databases. Systematic reviews and epidemiological studies from LMICs that included children aged one month to 9 years and reported outcomes of pneumonia with a penicillin-resistant pneumococcus in respiratory and blood culture specimens with or without comparison groups were included. Risk of bias was assessed using the Effective Public Health Practice Project (EPHPP) Quality Assessment Tool for Quantitative Studies. A narrative synthesis of findings based on the results of included studies was performed.

**Results:**

We included 7 articles involving 2864 children. One strong- and four medium-quality studies showed no difference in clinical outcomes (duration of symptoms, length of hospital stay and mortality) between those children with penicillin non-susceptible compared to susceptible pneumococci. Two weak quality studies suggested better outcomes in the penicillin-susceptible group.

**Conclusions:**

Current evidence suggests no difference in clinical outcomes of child pneumonia due to a penicillin-resistant *S. pneumoniae* and as such, there is no evidence to support a change in current WHO antibiotic guidelines.

*Streptococcus pneumoniae* is one of the most common bacteria causing pneumonia internationally and pneumococcal infections cause approximately 300 000 deaths in children under five years of age each year [1,2]. In the context of the global health threat of increasing antimicrobial resistance (AMR), surveillance for emerging resistance in *S. pneumoniae* isolates is required to prevent treatment failure and associated morbidity and mortality. AMR can be driven by misuse and overuse of antibiotics [3]. In regions with high (>50%) uptake of the pneumococcal conjugate vaccine, such as the Eastern Mediterranean, European, and African regions [4-6], there is evidence that the introduction of the 10- and 13-valent pneumococcal conjugate vaccine (PCV10 and PCV13) reduced rates of AMR, but these results were heterogeneous between studies likely reflecting the multiple drivers of AMR that differ between countries and over time [7]. Current World Health Organization (WHO) guidelines recommend penicillins as first-line treatment for childhood pneumonia [8], but this may need to be reviewed in the context of changes in pneumococcal AMR trends, particularly since the adoption of high valent (PCV10 or PCV13) in national childhood immunization programs.

In low- and middle-income countries (LMICs), the frequency of penicillin resistance based on non-meningitis breakpoints used for treatment of pneumonia ranges between 10% and 56% [9-12]. In the South East Asia region, a systematic review of studies from India found that 10% of invasive pneumococcal disease (IPD) isolates from children aged five years or younger were penicillin resistant [9]. Higher rates of penicillin resistance of 29% and 56% were found in two reviews of studies from China [10,11]. A systematic review from the South Asian Association for Regional Cooperation (SAARC) countries, involving seven countries from two WHO regions, including India, Pakistan, Bangladesh, Sri Lanka, Nepal, Bhutan, Maldives, and Afghanistan, found that 25% of IPD isolates were penicillin non-susceptible in children under five years [12]. A systematic review of antimicrobial resistance in Africa found penicillin resistance in *S. pneumoniae* was reported in 14/144 studies (with a median resistance of 26.7%), although this included studies involving all age-groups, with 24% of included studies involving paediatrics and neonates, 29% including both adults and paediatrics, and 42% including adults alone [13]. These studies demonstrate that penicillin-resistant pneumococci need to be considered in the treatment guidelines for children with pneumococcal infections in LMICs.

To determine the appropriate antibiotic choice in regions with higher rates of penicillin-resistant pneumococci it is critical to understand to what extent antibiotic resistance in vitro relates to poorer clinical outcomes. Therefore, this systematic review summarizes the literature on the clinical outcomes of pneumonia with penicillin-group resistant pneumococci in respiratory and blood culture specimens in children aged one month to 9 years in LMICs.

## METHODS

### Protocol and registration

This systematic review was registered on PROSPERO in Oct 2020. Registration number: CRD42020208192. No amendments to this registration were made.

### Eligibility criteria

Eligible studies included children aged one month to nine years in LMICs (as per World Bank income level) and reported clinical outcomes of pneumonia (including community acquired, nosocomial, and ventilator-associated) with a penicillin, ampicillin, amoxicillin and/or penicillin-group resistant pneumococcus in respiratory (either upper respiratory and/or lower respiratory tract) and/or blood culture specimens with or without comparison groups. English-language systematic reviews, observational and intervention studies, published between 2000 to the date of search were included. These dates were chosen in order to capture recent AMR prevalence trends, particularly since the introduction of PCVs. Literature reviews, case series, case reports and conference, meeting, and poster abstracts were excluded.

### Data sources

Four electronic databases were searched between September 2020 and November 2020 including MEDLINE, EMBASE, PubMed, and the Cochrane Library. For studies identified through trial databases that were still recruiting, or where recruitment status was unclear, the authors were contacted to provide clarification on study status and to determine whether relevant results were available.

### Search strategy

The search strategy included: terms relevant to the population of interest, including pneumonia and children; the exposure of interest, including pneumococcal infections and *S. pneumoniae*; and types of specimens such as respiratory samples and blood cultures. It also included an extensive list of terms related to the outcome of interest, antimicrobial resistance, and to the setting of interest, low and middle-income countries. See Appendix S1 in the **Online Supplementary Document** for the full MEDLINE search strategy.

### Study selection

Titles and/or abstracts of studies retrieved using the search strategy were screened independently by two reviewers (MHN, RL) to identify studies that met the eligibility criteria. The full text of potentially eligible studies was then independently assessed for inclusion by these two reviewers. Any disagreement that was not resolved between these two reviewers regarding study eligibility was resolved through discussion with a third reviewer (AG). This screening and selection process was performed using Covidence.

Two reviewers (MHN, RL) extracted data using a standardised table for assessment of study quality and evidence synthesis. Extracted information included: Study setting (country, WHO region); study design; participants; whether the study was done in a pre- or post-PCV vaccination era; definition of pneumonia used; AMR testing methodology, susceptibility breakpoints used; and clinical outcomes. CLSI susceptibility breakpoints for penicillin were divided into meningitis and non-meningitis cut-offs for *S. pneumoniae* with MIC values of ≤0.06 mg/L considered susceptible for meningitis and ≤2 mg/L for non-meningitis [14].

### Risk of bias in individual studies

Two review authors (MHN, RL) assessed the risk of bias using the Effective Public Health Practice Project (EPHPP) Quality Assessment Tool for Quantitative Studies [15].

### Synthesis of results

A narrative synthesis of findings based on the results of included studies was performed. A meta-analysis was not able to be performed due to the heterogeneity of clinical outcomes reported, and levels of bias of included studies.

## RESULTS

### Study selection

**Figure 1:**
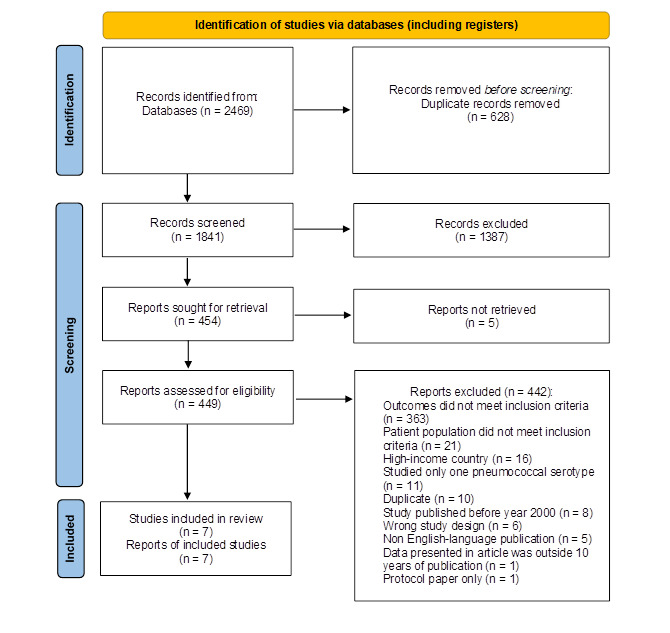
PRISMA 2020 Flow Diagram for Systematic Review.

Overall, the titles/abstracts of 1841 studies were screened and for 449 the full text was reviewed. Of these, 442 were excluded, with the most common reasons for exclusion being that the: outcomes (n = 363) and patient population did not meet inclusion criteria (n = 21); the study was conducted in a high-income setting (n = 16); or the study reported on only one pneumococcal serotype (n = 11). After full-text screening, seven studies were eligible for final inclusion. See **Figure 1**.

### Risk of bias

Of the seven included studies one was assessed as strong quality, four were moderate, and two were weak. See Table S1 in the **Online Supplementary Document** for EPHPP quality appraisal full scoring.

**Table 1** shows the characteristics and key results of included studies. All studies were either prospective or retrospective cohort studies, and all were set in a pre-PCV context. No relevant systematic reviews were identified. Overall, there were 2864 children included in the seven studies. The smallest study included 20 children with pneumonia [23], and the largest study involved 1215 children [18]. Four of the included studies were from the Region of the Americas [16,17,20,22], and there were two studies from the East Asia region [19,21] and one from Africa [18]. The prevalence of penicillin-resistant pneumococci ranged from 7% in a study from Peru [20] to 51% in a Mexican study [17]. Reported clinical outcomes included mortality (5 studies) [18-22], length of hospital stay (2 studies) [19,22], response to therapy (1 study) [17] or treatment failure (1 study) [16]. Three studies used the WHO definition of pneumonia [16,18,22], two used alternative definitions [17,20], and two did not provide the definition used [19,21].

**Table 1:** Characteristics and key results of included studies

Study	Country, region, study period	Study design	Participants	Pre- or post-PCV	Definition of pneumonia used	Method of AMR testing/ definition used	Prevalence of penicillin-group resistant pneumococci in specimens	Treatment received	Clinical outcomes	Key results	EPHPP rating
Cardoso, 2008 [16]	Multi-country, Region of the America, 1998-2002	Prospective observational cohort	240 children aged 3 to 59 m with CAP of which:	Pre-	As per WHO guidelines	Culture, then BMD/ S≤0.06 mg/L, I 0.12-1 mg/L, R≥2 mg/L*, NCCLS cut-off meningitis	263 pneumococcal isolates from BC and pleural fluid in 240 children:	IV Pen G 200 000 IU/kg/d or IV ampicillin 150 mg/kg/d	Treatment failure using clinical criteria†	% with treatment failure	Strong
			• 64 (27%) had PISP				• PSSP 138/263 (52%)			• Penicillin S 25/119 (21%)	
			• 120 (50%) had PSSP				• PISP 68/263 (26%)			• Pen I 14/63 (22%)	
			• 56 (23%) had PRSP				• PRSP 57/263 (22%):			• Pen R 10/54 (19%)	
							MIC 2 mg/L (n = 49)			*P*-value = 0.87‡	
							MIC 4 mg/L (n = 8)				
Gomez-Barreto 2000 [17]	Mexico/Region of the Americas, 1995-1999	Prospective observational cohort	49 children median age 16 m (range 1 m to 11 y) with severe IPD, 21 with pneumonia:	Pre-§	Pneumonia with signs of lower respiratory tract infection AND bacteremia OR recovery of pathogen from pleural fluid	DD and BMD/NCCLS, NCCLS cut-off meningitis	49 pneumococcal isolates from all sterile sites:	Either of: IV Pen G 100 000 IU/kg/d; PO amoxicillin 80 mg/k/d; IV ceftriaxone 100 mg/k/d; IV cefotaxime 100 mg/k/d ± IV vancomycin 40 mg/k/‖	Response to therapy	% who improved:	Weak
			• 6 (29%) had PRSP				• Pen R 25/49 (51%),			• PSSP 9/15 (60%)	
			• 15 (71%) had PSSP				IC≥2 mg/L (n = 14)			• PRSP 2/6 (33%)	
							MIC≥4 mg/L (n = 11)			*P*-value = 0.27‡	
Madhi 2000 [18]	South Africa/, Africa, 1997-1998	Prospective observational cohort	1215 children with CAP of which:	Pre-	WHO criteria for severe acute LRTI ± oxygen saturation <90% in room air,	BMD/ NCCLSC NCCLS cut-off meningitis	58 pneumococcal isolates in BC, susceptibility reported for 50:	Ampicillin or cefuroxime	Mortality	Results reported only for HIV + children PRSP not associated with a higher case fatality rate compared with PSSP:	Mod
			• 548 were HIV+ (median age 9 y, IQR 4-8)				• PRSP 23/50 (46%)			2/20 (10%) PRSP vs 4/17 (23.5%) PSSP; *P* = 0.38	
			• 617 were HIV- (median age 8y, IQR 4-14)								
Netsawang 2010 [19]	Thailand/South-East Asia Region, 1998-2007	Retrospective cohort	106 children median age 1 y (range 26d-13y) with IPD, 12 with pneumonia	Pre-	Not provided	Gradient plate/ CLSI NCCLS non-meningitis cut-off	106 pneumococcal isolates from sterile sites of which 75 non-meningitis:	Not stated	Mortality Length of stay	• PSSP: 9/56 (16%)	Mod
							• PSSP 56/75 (75%)			• PNSSP: 3/19 (16%)	
							• PRSP 19/75 (25%)			Mean length of stay (days): PSSP 14.3 (SD 16.8) vs PNSSP 11.3 (SD 10.1) (*P* = 0.47)	
Ochoa 2010 [20]	Peru/Region of the Americas, 2006-2008	Prospective observational cohort	101 episodes of IPD in 97 children median age of 1.2 y (range 1 m-15.8 y), 48 with pneumonia	Pre-	Positive blood or pleural fluid culture AND fever, respiratory distress AND pulmonary infiltrates on chest x-ray.	Optochin susceptibility and E-test/, CLSI/NCCLS, NCCLS non-meningitis cut-off	101 pneumococcal isolates from sterile sites, of which 62 were non-meningitis:	Not stated	Mortality	No difference proportion of children with PNSSP in fatal vs non-fatal group: 4/20 (20%) fatal vs 15/71 (21%), *P* = 0.91‡	Mod
							PSSP 57/62 (92%)				
							• PISP 1/62 (1%)				
							• PRSP 4/62 (7%)				
Pancharoen 2001 [21]	Thailand/ South-East Asia Region, 1986-1997	Retrospective cohort	68 patients including20 children mean age 4 y (range 3 m to 15 y) with pneumonia	Pre-	Not provided	DD and E-test in 8/17 strains/R≥0.12 mg/LNCCLS cut-off meningitis	19 pneumococcal isolates from sterile sites from children with pneumonia:	Not stated	Mortality	0/20 with pneumonia	Mod
							• PSSP 16/19 (84%)				
							• PRSP: 3/19 (16%)				
Pirez 2001 [22]	Uruguay/Regional office for the Americas, 1997-1998	Prospective nested cohort study	1163 children aged <1 m to 5 y with CAP	Pre-	For <5 y WHO/PAHO criteria used. Classic clinical and radiologic findings for older children.	E-test/ S≤0.06 mg/L. I≥0.12-1 mg/L, R≥2 mg/L, NCCLS cut-off meningitis	41 pneumococcal isolates from BC and/or pleural fluid:	Aged 1 m-5 y: IV ampicillin 300mg/kg/d, Aged >5 y: IV pen G 200 000 IU/kg/d	Admission to ICU Mortality Length of stay (% with >14 d in hospital)	PSSP 6/30 (20%)	Weak
							• PSSP 30/41 (73%)			PISP 0/6 (0%)	
										PRSP 3/5 (60%)	
										*P*-value = 0.07‡	
										PSSP 1/30 (3%)	
										PISP 0/6 (0%)	
							• PISP 6/41 (15%)			PRSP 1/5 (20%)	
										*P*-value = 0.25‡	
										PSSP 16/30 (53%)	
										PISP 2/6 (33%)	
										PRSP 3/5 (60%) *P*-value = 0.69‡	
							• PRSP: 5/41 (12%)				

BMD – broth micro–dilution, DD – disk diffusion, NCCLSC – National Committee for Clinical Laboratory Standards criteria, NM – non–meningitis, CLSI – clinical and laboratory standards institute, PSSP – penicillin susceptible streptococcus pneumoniae, PNSP – penicillin non–susceptible streptococcus pneumoniae, PISP – penicillin intermediate susceptible pneumococcus, S – susceptible, I – intermediate, R – resistant, CAP – community–acquired pneumonia, HAP – hospital–acquired pneumonia, BC – blood culture, PF – pleural fluid, IV – intravenous, PO – orally, CFR – case fatality rate, Mod – moderate, d – days, m – months, y – years, IQ – interquartile, ABx – antibiotics, IPD – invasive pneumococcal disease, PAHO – Pan American Health Organization

*CLSI/NCCLS standards.

†Defined as either no improvement (persistence of fever, tachypnoea, dyspnoea or hypoxemia) after at least 48 h of ABx therapy or deterioration of patients during antimicrobial therapy.

‡Calculated using Fisher exact test on STATA 16.1 (StataCorp LLC, Lakeway Drive College Station, TX, USA)

§Mexico introduced PCV7 to NIP in 2006. Reference: Wasserman M, Palacios MG, Grajales AG, Baez/Revueltas FB, Wilson M, McDade C, et al. Modelling the sustained use of the 13-valent pneumococcal conjugate vaccine compared to switching to the 10-valent vaccine in Mexico. Human vaccines & immunotherapeutics.

‖8 children received vancomycin, but unsure who of the participants with pneumonia received this.

¶This included both 1/19 isolates that was resistant on oxacillin disc diffusion testing (MIC not performed), and 2/19 that had MIC≥0.12 µg/mL by E-test.

Regarding the laboratory methods used to determine AMR, three studies used broth microdilution [16-18] which is the gold standard method for determining the minimum inhibitory concentration (MIC) of bacteria [24], and the remaining studies used disc diffusion (**Table 1**). Two studies used the Clinical Laboratory Standard Institute (CLSI) methodology [16,17], two referenced alternative methodology [19,22], and three did not state the methods used [18,20,21]. Of the seven included studies, only two used non-meningitis breakpoints appropriate for pneumonia [19,20], three used meningitis breakpoints [16,21,22], and two did not state which breakpoints were used [17,18]. The antibiotic treatment received was described in only three studies [16,17,22], and doses of penicillin G ranged from 100 000 to 200 000 IU/kg/d, while ampicillin doses ranged from 150 to 300 mg/kg/d.

Of the included studies, the only study assessed as strong quality was a multi-country prospective cohort study that reported no difference in treatment failure between children with penicillin-susceptible and non-susceptible pneumococci (susceptible 21%, intermediate 22%, resistant 19%, *P* = 0.87) [16]. However, this study used the susceptibility breakpoints for meningitis and of the penicillin-resistant pneumococci, 49/57 (86%) would have been classed as susceptible according to the non-meningitis breakpoints (≤2 mg/L), and it was not possible to re-analyse the databased on this as there was insufficient linkage of resistant pneumococci’s corresponding MICs to clinical outcomes in the reported data.

The four moderate quality studies all reported no difference in clinical outcome in those with penicillin-susceptible and non-susceptible pneumococci. A retrospective cohort study from Thailand found no increase in mortality or length of stay in those with non-susceptible *S. pneumoniae* isolates using non-meningitis breakpoints [19]. However, this study only included 12 children with pneumonia and clinical outcomes were reported for all children with IPD rather than those specifically with pneumonia [19]. Another study also found no increase in mortality in a prospective cohort study of 101 children with IPD of whom 48 had pneumonia [20]. Neither of these studies stated the treatment received. One study that included children with HIV infection, found that infection with penicillin-resistant pneumococci was not associated with a higher case fatality rate compared with penicillin-susceptible strains, however these children may have been treated with either ampicillin or cefuroxime [18]. Another study similarly found that no deaths occurred in 19 children with either penicillin-resistant or susceptible pneumococcal infections [21]. Of note, of the latter three studies, only one used the non-meningitis breakpoints to define susceptibility [18} and for the remaining two, the breakpoints were not stated.

The two studies assessed as weak quality reported variable results, with one study finding that fewer children with pneumonia due to penicillin-resistant pneumococci improved compared to those with a susceptible strain (resistant 2/6 (33%) vs susceptible 9/15 (60%)) [17]. Similarly, in a retrospective study of 1163 children, those with penicillin-resistant *S. pneumoniae* were more frequently admitted to the intensive care unit, although this difference was not significant (*P* = 0.07), potentially due to a small sample size of children with penicillin non-susceptible S. *pneumoniae* [22]. In this study, a higher frequency of pneumonia complications were observed in those with penicillin-resistant pneumococci compared with penicillin-susceptible species (empyema 4/5 (80%) vs 19/30 (63%), *P* = 0.47; pneumatocele 4/5 (80%) vs 10/30 (33%), *P* = 0.05), however this difference was not significant for empyema, with some evidence for pneumatocele [22]. Gomez-Barreto 2000 did not state what MIC breakpoints were used and Pirez 2001 used meningitis breakpoints [22].

### Synthesis of results

Given that only one study was assessed as being of strong quality, and the lack of consistent reporting of clinical outcomes across studies, a meta-analysis was not performed.

## DISCUSSION

This review of the clinical outcomes of pneumonia with a penicillin-group resistant pneumococcus in respiratory and blood culture specimens in children aged one month to nine years in LMICs identified seven heterogeneous studies of predominantly moderate or low quality. Based on the existing literature from the pre-PCV era, evidence suggests that the outcomes of children with penicillin non-susceptible pneumonia are not significantly worse than those who are infected with a susceptible strain and therefore there is no evidence to support a change in the current WHO treatment approach to pneumonia.

The outcomes of this review must be interpreted cautiously because of the inconsistency in the definitions of pneumonia used and the outcome reporting across the studies, limited data on children specifically with pneumonia, all studies being from a pre-PCV era and finally, that in only two studies were the appropriate non-meningitis susceptibility breakpoints used. Another key limitation was overall poor study quality, with only one of the final studies assessed as strong. Almost all studies did not adjust for confounding, making the interpretation of these results difficult.

Most studies included in this systematic review looked at pneumococci isolated from the gold standard, sterile site specimens such as blood cultures or pleural fluid. AMR prevalence data are usually captured through routine vaccine-preventable disease surveillance, or through nasopharyngeal (NP) carriage surveys. However, it is very difficult in many settings to have sufficient invasive isolates to adequately monitor AMR, so NP AMR surveys are undertaken and used as a proxy for invasive disease AMR. Studies suggest that the correlation between AMR in pneumococci isolated from the upper vs lower respiratory tract and/or blood culture specimens in children with pneumonia is variable. A prospective study of children with acute lower respiratory tract infections found that the serotype and antimicrobial susceptibility of all 85 S. *pneumoniae* isolates from throat swabs were the same as those identified from blood cultures [25]. Similarly, the AMR-profile of pneumococcal isolates from NP swabs in children aged 12 months of age correlated with those from hospital-based surveillance data from a range of specimens [26]. In contrast, higher rates of resistance from invasive isolates (2/35, 6%) compared to NP specimens (20/1013, 2%) were reported in a study of paired NP and lower respiratory tract specimens and/or blood culture samples from the Philippines [27]. Similarly, a study from South Africa that compared NP swabs with sputum and/or bronchoalveolar lavage (BAL) samples from children with pneumonia found that 4/6 (67%) sputum/BAL samples were penicillin-resistant compared to 12/28 (43%) in NP samples [28]. It is, therefore, unclear whether AMR findings from upper airways specimens should be used to guide antimicrobial treatment in children with pneumonia. However, in areas where the prevalence of penicillin-resistance is high, it would be prudent to closely monitor the clinical status of the patient and may consider switching to an alternative therapy to cover penicillin-resistant pneumococci in severely ill patients (eg, ceftriaxone, vancomycin).

A key limitation of this review is that only four of the seven included studies reported the antibiotic treatment received, although we expect these study sites followed WHO treatment guidelines. Also most studies did not specifically look at the relationship between resistance and severe pneumonia, although one study showed evidence to support increased risk of pneumatocele in children with penicillin-group resistant pneumococcus [22]. Additionally, there was significant variability in therapy with some studies using antibiotics indicated for the treatment of penicillin-resistant pneumococcus (ie, cephalosporins and vancomycin), and therefore it is difficult to determine the effectiveness of penicillin-based therapy on clinical outcomes for penicillin-resistant pneumococcal pneumonia for these studies. Another limitation is that none of the included studies were from the Eastern Mediterranean, Western Pacific, or LMICs from the European WHO regions. Moreover, only studies from Thailand were included from the South East Asia region. This means that there was no data from countries with the highest burden of pneumonia such as India, Nigeria, Indonesia, Pakistan and China [1]. In addition to having a high burden of pneumonia, countries from the South East Asia region may be more at risk of antibiotic resistance due to limited antibiotic stewardship and the wide availability of antibiotics without a prescription [29]. As well as this lack of regional presentation, all final studies were set in the pre-PCV era, making it difficult to generalize to countries that have adopted higher-valent PCVs in their national immunization programs. A systematic review published in 2019 studied the impact of high-valent PCV (PCV10 & PCV13) on AMR and showed that PCV13 reduced the rates of AMR in both invasive isolates and NP samples from children with IPD [7]. In this review, only three studies examined the impact of PCV10 on AMR, with two studies set in Brazil finding a reduction in the incidence of penicillin-resistant IPD, another study from Finland finding no change [7]. This suggests that the rate of AMR may be lower in these regions. Our review found that while there was modest evidence of reductions in AMR following the introduction of PCV10 or PCV13 there was some heterogeneity in these findings (unpublished results). Routine surveillance systems are needed to detect emergent penicillin-resistance among pneumococcal isolates, particularly in areas with high antibiotic use and misuse.

In this review doses of IV ampicillin varied between 150 to 300 mg/kg/d, and for oral amoxicillin, one study used a dose of 80 mg/kg/d. The WHO IMCI Blue book recommends an amoxicillin dose of at least 40 mg/kg twice daily (ie, 80 mg/kg/d) for non-severe pneumonia (non-hospitalised) [8]. These are similar to the recommendations from the American Academy of Paediatrics and the American Academy of Family Physicians for the treatment of community-acquired pneumonia that suggest a dose of 75 to 100 mg/kg/d [30]. For β-lactam antibiotics such as amoxicillin, clinical success is predicted by the time above which the free drug concentration exceeds the bacterial MIC with the target being greater than 40%-50% of the dosing interval [31]. Therefore, more frequent dosing is required to overcome penicillin-resistance for pneumococcus [32], and there is evidence that IV doses of penicillin ranging from 100 000 to 300 000 U/kg per day in 4-6 divided doses is effective [33]. A pharmacokinetic study of amoxicillin + clavulanic acid found that free amoxicillin levels exceeded an MIC of 8 mg/L for at least 40% of the dosing interval with doses of 25 mg/kg 4, 6, or 8 hourly in children older than 3 months of age and 25 mg/kg 12 hourly in children aged 1 to 3 months [34]. However, increasing the frequency of dosing must be weighed against treatment adherence due to the higher medication burden and for IV antibiotics, increased staff workload. Furthermore, amoxicillin has been shown to concentrate in bronchial mucosa and therefore, even at low doses can achieve high concentrations in lung tissue. This may explain why no significant clinical difference was observed in children with penicillin-resistant pneumococcal pneumonia in this review [35].

Future studies should be specifically designed to detect the association between penicillin-resistant *S. pneumoniae* and clinical outcomes for pneumonia in the post-PCV era using breakpoints for non-meningitis. This should be incorporated into IPD and NP carriage surveillance systems. Research that evaluates higher doses of first line antibiotics in LMIC settings should also be performed. Surveillance data can also be used to identify areas with a high prevalence of AMR for future research evaluating the use of alternatives compared to penicillin therapy for empirical treatment of pneumonia in children.

## CONCLUSIONS

Our review found that the majority of studies did not show that children with pneumonia due to a penicillin-resistant *S. pneumoniae* have an increased mortality or longer length of hospital stay compared to those with a penicillin-susceptible strain. Therefore, there is insufficient evidence to support a change in current WHO antibiotic guidelines for the care of children with pneumonia. However, the published studies to date have major limitations, and this recommendation is primarily based on weak or moderate-quality evidence. This study highlights the importance of the routine collection of data on penicillin-susceptibility using non-meningitis and meningitis breakpoints as part of national surveillance systems.

## Additional material


Online Supplementary Document
