# Supplementary material for: Systematic review of the clinical outcomes of pneumonia with a penicillin-group resistant pneumococcus in respiratory and blood culture specimens in children in low- and middle-income countries
Source: J Glob Health. 2022 Aug 22;12:10004. doi: 10.7189/jogh.12.10004 (PMC9393747; doi:10.7189/jogh.12.10004)
Supplement: Online Supplementary Document [file jogh-12-10004-s001.pdf]

Appendix S1: MEDLINE Search Strategy

1. exp Pneumonia/di, dt, ep, mi
2. Pneumococcal Infections/di, dt, ep, mi
3. ((lower-respiratory-tract adj3 infection*) or acute-respiratory-infection* or invasive-pneumococcal-disease* or parapneumonic or para-pneumonia or pneumonia or pneumonias or lung-inflammation* or lobitis or nonspecific-inflammatory-lung-disease* or peripneumonia or pleuropneumonia or pleuropneumonitis or pneumonic-lung* or pneumonic-pleurisy or pneumonic-pleuritis or pneumonitides or pneumonitis or pulmonal-inflammation* or pulmonary-inflammation* or pulmonic-inflammation*).tw,kf.
4. Bacteremia/di, dt, ep, mi
5. exp empyema, pleural/di, dt, ep, mi or exp pleural effusion/di, dt, ep, mi
6. Streptococcus pneumoniae/
7. (bacter?emia or empyema or pleural or bronchial or bronchoalveolar or alveolar or endotracheal or tracheal).tw,kf.
8. (pneumoniae or pneumococc*).tw,kf,hw.
9. (4 or 5 or 7) and (6 or 8)
10. 1 or 2 or 3 or 9
11. Anti-Bacterial Agents/pd
12. exp Penicillins/
13. Penicillin*.tw,kf.
14. (a-gram or abdimox or acilina or acimox or actimoxi or adbiotin or agerpen or alfamox or alfoxil or almodan or almorsan or alphamox or amagesen-solutab or ameclina or amitron or amo-flamisan or amo-flamsian or amocillin or amoclen or amodex or amoflux or amohexal or amolin or amonex or amopen or amophar-ge or amosine or amoval or amoxa or amoxal or amoxapen or amoxaren or amoxcil or amoxcillin or amoxcin or amoxi-basan or amoxicilina or amoxicilline or amoxiclin or amoxicot or amoxidal or amoxidin or amoxidrops or amoxihexal or amoxil or amoxillin or amoxina or amoxipen or amoxipenil or amoxisol or amoxivan or amoxivet or amoxy or amoxycillin or amoxycilline or ampliron or apo-amoxi or ardine or aroxin or azillin or bacihexal or bactamox or bactox-ge or beamoxy or betamox or bimox or bintamox or biomox or biotamoxal or bioxidona or bioxyllin or bristamox or brl-2333 or brl2333 or broadmetz or cabermox or cilamox or clamox or clamoxyl or clearamox or clonamox or coamoxin or damoxicil or dispermox or doxamil or draximox or edamox or efpinex or erphamoxy or eupen or farconcil or fisamox or flemoxin or flemoxine-ge or fluamoxina or foxolin or fullcilina or gexcil or gimalxina or glamox or glassatan or gomcillin or grinsul or grunamox or hamoxillin or hiconcil or hidramox or hipen or hosboral or ibamox or ibiamox or ikamoxil or imacillin or imaxilin or inamox or infectomycin or intermox or isimoxin or izoltil or julphamox or jutamox or kamoxin or ladoxillin or lamoxy or larocilin or larocin or larotid or macromox or magnimox or maxamox or maxcil or medimox or meixil or metifarma or mopen or morgenxil or moxacin or moxaline or moxarin or moxilen or moxilin or moximar or moxitab or moxtid or moxylin or moxypen or moxyvit or neogram or novabritine or novamox or novamoxin or novenzymin or novoxil or nuvosyl or optium or oramox or ospamox or pamocil or pamoxicillin or pamoxin or panvilon or pasetocin or penamox or penbiosyn or pentyloxycillin or pharmoxyl or piramox or polymox or pondnoxcill or rancil or ranmoxy or ranoxil or ranoxyl or

robamox or romoxil or ronemox or saltermox or sawacillin or sawamezin or servamox or shamoxil or siamox or sigamopen or sil-a-mox or silamox or simoxil or sintopen or solamocta or solpenox or sumox or superpeni or teramoxyl or tolodina or tormoxin or triafamox or triamoxil or trifamox or trimox or uro clamoxyl or uroclamoxyl or utimox or vastamox or velamox or vistrep or widecillin or winpen or wymox or xiltrop or zamocillin or zamox or zamoxil or zerrsox or zimox).tw,kf.
15. (acillin or aldribid or aletmicina or alfasilin or alpha-aminobenzylpenicillin or alphacin or ambiopi or amblocin or amblosin or amcill or amcillin or amficot or amfipen or aminobenzylpenicillin or amipenix or amoxi or amoxine or ampcillin or ampecu or ampen or ampenolet or ampensaar or ampexin or ampibex or ampiblan or ampicher or ampicil or ampicilin or ampicilina or ampiciline or ampicillin or ampicilline or ampicin or ampicyn or ampidar or ampifen or ampiflex or ampiger or ampilag or ampillin or ampimedin or ampipen or ampitenk or ampivral or ampkid or amplacilina or amplibin or ampliblan or amplital or amplivacil or ampolin or ampycin or amsapen or anglopen or anhypen or apo-ampi or austrapen or ay-6108 or ay6108 or bayer-5427 or binotal or biocil or bremcillin or bridopen or britapen or brl-1341 or brl1341 or c-10575 or c10575 or camicil or cetampin or cimexillin or citicil or clovillin or copharcilin or dhacillin or diferin or doktacillin or doltirol or domicillin or dotirol or duacillin or dumopen or eracillin or eurocin or excillin or extrapen or fontapen or gramcil or h-ambiotico or helvecillin or herpen or hi-63 or hi63 or hostes or ibimycin or ikacillin or intramed or iwacillin or jenampin or julphapen or ks-r1 or marticil or mecil-n or neosensitabs or nuvapen or omnipen or p-50 or pamecil or panacta or pen-an or penbristol or penbritin or penbritin-s or penicline or penodil or penstabil or pentrex or pentrexil or pentrexyl or petercillin or pfizerpen-a or picylin or polycillin or polyflex or polypen or pricillin or primapen or princillin or principen or radiocillina or redicillin or rimacillin or roscillin or semicillin or servicillin or shacillin or sintelin or standacillin or standcillin or synpenin or synthocilin or synthocillin or tolimal or totacillin or totapen or trafarbiot or tricil or trifalicina or trihypen or trilaxin or ukapen or usampi or vacillin or viccillin or vidopen or virucil or vitapen).tw,kf.
16. 11 or 12 or 13 or 14 or 15
17. exp Microbial Sensitivity Tests/
18. exp Drug Resistance, Microbial/
19. (resistance or resistant or susceptib* or sensitivit* or nonsusceptib* or minimum-inhibitory-concentration*).tw,kf.
20. 17 or 18 or 19
21. developing countries/
22. (austere or (limited adj2 resource*) or (low adj2 resource*) or (transitioning adj econom*) or (third adj world) or LMIC or LMICs or (lami adj countr*) or (transitional adj countr*) or (low adj gdp) or (low adj gnp) or (low adj gross adj domestic) or (low adj gross adj national) or ((emerging or developing or (low adj income) or (middle adj income) or (low adj3 middle) or underdeveloped or under-developed or (less* adj developed) or underserved or under-served or deprived or poor*) and (countr* or nation*1 or econom* or population or world))).tw,kf.
23. exp africa/
24. americas/ or exp caribbean region/ or exp central america/ or latin america/ or mexico/ or exp south america/
25. europe/ or exp europe, eastern/ or exp transcaucasia/
26. antarctic regions/ or exp atlantic islands/ or exp indian ocean islands/ or exp pacific islands/

27. New Guinea/
28. asia/ or exp asia, central/ or asia, southeastern/ or borneo/ or cambodia/ or east timor/ or indonesia/ or laos/ or malaysia/ or mekong valley/ or myanmar/ or philippines/ or thailand/ or vietnam/ or asia, western/ or bangladesh/ or bhutan/ or india/ or middle east/ or afghanistan/ or iran/ or iraq/ or jordan/ or lebanon/ or oman/ or saudi arabia/ or syria/ or turkey/ or yemen/ or nepal/ or pakistan/ or systematic reviewi lanka/ or far east/ or china/ or tibet/ or exp korea/ or mongolia/
29. (Afghanistan or Albania or Algeria or Angola or Antigua or Argentina or Armenia* or Aruba or Azerbaijan or Bahrain or Bangladesh or Barbados or Barbuda or Belarus or Byelarus* or Byelorussian or Belorussian or Belorus* or Belize or Benin or Bhutan or Bolivia or Bosnia or Botswana or Brasil or Brazil or Bulgaria or (Burkina adj Fas*) or (Upper adj Volta) or Burma or Burundi or Cambodia or Khmer or Kampuchea or Cameron* or Cameroon* or (Cape adj Verde) or (Cabo adj Verde) or (Central adj African adj Republic) or Chad or Chile or China or Colombia or Comoros or (Comoro adj Island*) or Comores or Mayotte or Congo or Kongo or (Cook adj Island*) or (Costa adj Rica) or (Cote adj D'ivoire) or Croatia or Cuba or Cyprus or Czech* or Djibouti or Dominica or Dominican or (East adj Timor) or (East adj Timur) or Ecuador or Egypt or El-Salvador or (Equatorial adj Guinea) or Eritrea or Estonia or Ethiopia or Fiji or (French adj Somaliland) or Futuna or Gabon or (Gabonese adj Republic) or Gambia or Gaza or (Georgia* adj Republic) or Ghana or Grenada or Guam or Guatemala or Guinea or Guiana or Guyana or Haiti or Herzeg* or Hercegovina or Honduras or Hungary or India or Indonesia or Iran or Iraq or (Ivory adj Coast) or Jamaica or Jordan or Kazakh* or Kenya or Kiribati or Korea or Kosovo or (Kyrgyz adj Republic) or Kyrgyzstan or Kirghizia or Kirghiz or Kirgizstan or Laos or (Lao* adj2 Democratic adj Republic) or (Lao* adj PDR) or Latvia or Lebanon or Lesotho or Basutoland or Liberia or Libya or Lithuania or Macedonia or Madagascar or (Magalasy adj Republic) or Malawi or Malay* or Sabah or Sarawak or Maldives or Mali or (Marshall adj Island*) or Mauritania or Mauritius or (Agalega adj Island*) or Mexico or Micronesia or Moldov* or Mongolia or Montserrat or Montenegro or Morocco or Ifni or Mozambique or Myanma* or Namibia or Nauru or Nepal or (Netherlands adj Antilles) or (Dutch adj Antilles) or (New adj Guinea) or (New adj Caledonia) or Nicaragua or Niue or Niger or Nigeria or (Northern adj Mariana adj Island*) or Nyasaland or Oman or Pakistan or Palau or Panama or (Papua adj New adj Guinea) or PNG or Palestine or Paraguay or Peru or Philipines or Philippines or Phillipines or Phillippines or Poland or (Puerto adj Rico) or Yemen or Romania or Roumania or Rumania or Russia* or Rwanda or Ruanda or (Saint adj Kitts) or (St adj Kitts) or Nevis or (Saint adj Vincent) or (St adj Vincent) or Grenadines or Samoa* or (Navigator adj Island*) or (Saint adj Lucia) or (St adj Lucia) or (Saint adj Helena) or (St adj Helena) or (Sao adj Tome) or (Saudi adj Arabia) or Senegal or Serbia or Seychelles or (Sierra adj Leone) or Slovenia or Slovak* or (South adj Africa) or (Solomon adj Island*) or Somalia or (Systematic reviewi adj Lanka) or Ceylon or Sudan or Surinam* or Swaziland or Syria or Tajikistan or Tadzhikistan or Tadjikistan or Tadzhik or Tanzania or Thailand or Tibet or Timor-Leste or Togo or (Togolese adj Republic) or Tokelau or Tonga or Trinidad or Tobago or Tunisia or Turkey or Turkmenistan or Turkmen or Tuvalu or Uganda or Ukraine or Uruguay or Urundi or USSYSTEMATIC REVIEW or (Soviet adj Union) or "Union of Soviet Socialist Republics" or Uzbekistan or Vanuatu or (New adj Hebrides) or Venezuela or Vietnam or (Viet adj Nam) or (Wallis adj2 Futuna) or (United adj Arab adj Republic) or (West adj Bank) or (West adj Indies) or Yemen or Yugoslavia or Zaire or Zambia or Zimbabwe or Rhodesia).tw,kf.
30. (africa or americas or caribbean or (central adj America) or (latin adj America) or (south adj America) or (eastern adj Europe) or Transcaucasia or antarctic or (atlantic adj island*) or (indian adj ocean adj

island*) or (pacific adj island*) or polynesia or (central adj asia) or (southeast* adj asia) or (south-east* adj asia) or borneo or mekong or (western adj asia) or (middle adj east) or (far adj east)).tw,kf.

31. 21 or 22 or 23 or 24 or 25 or 26 or 27 or 28 or 29 or 30

32. (newborn* or new-born* or baby or babies or neonat* or neo-nat* or infan* or toddler* or pre-schooler* or preschooler* or kinder or kinders or kindergarten* or kinder-aged or boy or boys or girl or girls or child or children or childhood or pediatric* or paediatric* or school-age* or schoolage* or schoolchild* or schoolgirl* or schoolboy*).af.

33. 10 and 16 and 20 and 31 and 32

34. (exp animals/ or (rat or rats or mouse or mice or swine or porcine or murine or sheep or lamb or lambs or pig or pigs or piglet or piglets or rabbit or rabbits or cat or cats or dog or dogs or cattle or bovine or monkey or monkeys or trout or marmoset or marmosets).ti.) not human*.sh.

35. 33 not 34

36. limit 35 to english language

Table S1. EPHPP Quality Assessment Full Scoring

Author	Selection bias	Study design	Confounders	Blinding	Data collection methods
**Cardoso**	Moderate	Moderate	Strong	Moderate	Strong
Madhi 2000	Moderate	Moderate	**Weak**	Moderate	Strong
Netsawang 2010	Strong	Moderate	Weak	Strong	Strong
Ochoa 2010	Moderate	Moderate	Weak	Moderate	Strong
Pancharoen 2001	Moderate	Moderate	Weak	Moderate	Strong
Pirez 2001	Weak	Moderate	Weak	Moderate	Strong
Gomez-Barreto 2000	Moderate	Moderate	Weak	Weak	Storng
